# Development of a Quantitative PCR Method for Detecting *Enterococcus faecalis* Cytolysin in Human Stool Samples

**DOI:** 10.3390/mps6060107

**Published:** 2023-11-08

**Authors:** Noemí Cabré, Yongqiang Yang, Yanhan Wang, Bernd Schnabl

**Affiliations:** 1 Department of Medicine, University of California San Diego, La Jolla, CA 92093, USA; ncabrecasares@ucsd.edu (N.C.); yoy006@health.ucsd.edu (Y.Y.); yaw015@health.ucsd.edu (Y.W.); 2 Department of Medicine, VA San Diego Healthcare System, San Diego, CA 92161, USA

**Keywords:** qPCR, *Enterococcus faecalis*, cytolysin, fecal samples, microbiota

## Abstract

Alcohol-associated liver disease (ALD) is a major global health issue, contributing significantly to morbidity and mortality worldwide. Among the ALD subtypes, alcohol-associated hepatitis poses a severe and urgent medical challenge with high short-term mortality rates. Despite extensive research, the current therapeutic approaches for alcohol-associated hepatitis have limited efficacy, necessitating novel interventions. Recent studies have highlighted the crucial role of the gut microbiota in ALD pathogenesis, particularly *Enterococcus faecalis* (*E. faecalis*) and its cytolysin exotoxin. This study presents the development of a standardized real-time quantitative polymerase chain reaction (RT-qPCR) assay to detect and quantify cytolysin in fecal samples from patients with alcohol-associated hepatitis. The diagnostic assay allows for an association analysis between cytolysin-positive *E. faecalis* and disease severity as well as mortality. This assay was developed to standardize the identification of cytolysin-positive patients who can be selected for clinical trials.

## 1. Introduction

Alcohol-associated liver disease (ALD) remains an important public health challenge worldwide, contributing significantly to global morbidity and mortality [1]. Cirrhosis, a severe consequence of chronic alcohol abuse, affects over 26 million individuals globally, imposing a substantial burden on healthcare systems [2]. Genetic, epigenetic, and non-hereditary factors are essential markers and risk predictors for this disease, emphasizing their critical role in the multifaceted nature of ALD. This encompasses not only the direct effects of alcohol but also the genetic and epigenetic influences on its development and progression [3].

Among the distinct entities that comprise ALD, alcohol-associated hepatitis emerges as a particularly grave and acute liver disease with cholestasis, characterized by high morbidity and short-term mortality rates. Despite considerable research efforts, the current therapeutic strategies for alcohol-associated hepatitis have yielded limited success, creating a dire need for novel and effective treatment approaches [4].

The human microbiota consists of a diverse array of bacteria, viruses, and fungi, each contributing significantly to various aspects of human health and the development and advancement of distinct diseases. Hence, maintaining a delicate balance between the host and these microorganisms is of paramount importance [5,6]. Emerging evidence highlights the pivotal role of the gut microbiota in the pathogenesis of various liver diseases, including ALD. Intestinal dysbiosis, characterized by a disruption in the balance of beneficial and pathogenic microbes, has been implicated in disease progression [7]. Notably, patients with ALD exhibit significant alterations in their gut microbiota composition, encompassing changes in bacterial, fungal, and viral communities [8]. Within this microbial ecosystem, *Enterococcus faecalis* (*E. faecalis*) and its two-subunit exotoxin, cytolysin, have recently emerged as compelling candidates with potential clinical implications [9]. Recent studies [9,10] observed an increased relative abundance of *E. faecalis* in the feces from patients with alcohol-associated hepatitis, specifically a strain that secretes the exotoxin cytolysin. The presence of cytolysin-positive *E. faecalis* correlated with the severity of liver disease and mortality rate in patients with alcohol-associated hepatitis [9]. The oral administration of cytolysin-positive *E. faecalis* promotes ethanol-induced liver injury in mice [9]. These findings suggest that cytolysin-positive *E. faecalis* may play a significant role in the development and progression of alcohol-associated hepatitis. However, the levels of cytolysin-positive *E. faecalis* do not serve as a predictive factor for disease severity in cases of acutely decompensated cirrhosis and acute-on-chronic liver failure nor in patients with non-alcoholic steatohepatitis [11,12], indicating that it could be a specific biomarker for alcohol-associated hepatitis.

The purpose of this summary protocol is to provide an overview of the development of a real-time quantitative polymerase chain reaction (RT-qPCR) assay designed for detecting and quantifying cytolysin, the exotoxin produced by *E. faecalis*, in the human gut. By utilizing advanced molecular techniques, this assay was developed to standardize the identification of cytolysin-positive patients who can be selected for clinical trials.

## 2. Experimental Design

Please note, all supplies and reagents used need to be sterile and DNA and RNA free.

### 2.1. Materials for Bacterial DNA Isolation from Stool

1. 0.5 mm Zirconium Oxide Beads (Next Advance, Raymer town, NY, USA, Cat. No.: ZROB05)
2. Absolute ethanol
3. Sterile 2 mL Screw-Cap Tubes (Stellar scientific, Baltimore, MD, USA, Cat. No.: T20-C3220-SG)
4. QIAmp Fast DNA stool Mini Kit (QIAGEN, Hilden, Germany; Cat. No.: 51604)
5. Pipettes and sterile tips
6. Eppendorf tubes 1.5 and 2 mL

### 2.2. Isolation of Enterococcus Strains

1. *Enterococcosel* Broth (BD Biosciences, La Jolla, CA, USA, Cat. No.: 212207)
2. Agar (BD Biosciences, La Jolla, CA, USA, Cat. No.: 214010)
3. Blood agar plates (BD Biosciences, La Jolla, CA, USA, Cat. No.: B21739X)
4. 4 mm glass sterile beads (Fischer Scientific, Waltham, MA, USA, Cat. No.: 11-312B)

### 2.3. Materials for qPCR

1. Primer mix (forward and reverse) for bacteria (see Primers at Procedure 3.2)
2. Genomic DNA (10 ng/μL)
3. MicroAmp Fast 96-Well Reaction Plate (0.1 mL) (Applied Biosystems, Waltham, MA, USA, Cat. No.: 4346907)

### 2.4. Materials for Gel Electrophoresis

1. Agarose gel (Invitrogen, Waltham, MA, USA, Cat. No:.16500500)
2. DNA ladder 100 bp (Biopioneer Inc. San Diego, CA, USA, Cat. No.: MDL-100)
3. SYBR^®^ Safe (Invitrogen, Waltham, MA, USA, Cat. No.: S33102)
4. TAE buffer (Quality Biological Inc, Gaithersburg, MD, USA, Cat. No.: 10128-398)

### 2.5. Equipment

- Centrifuge (capacity to speed at 20,000× *g*)
- Nanodrop
- Vortex
- Heat blocks
- Mini-Beadbeater 96, BioSpec Products (capacity to speed at 2000 rpm)
- Applied Biosystems^TM^ StepOnePlus^TM^ real-time PCR system
- Gel Electrophoresis Equipment

### 2.6. Methods for Human Studies, Mouse Studies, and Statistics

#### 2.6.1. Patient Cohorts

Patients with alcohol-associated hepatitis were recruited from the InTeam Consortium across various countries, including the USA, Mexico, Canada, UK, France, and Spain. The inclusion criteria mandated recent active alcohol abuse (more than 50 g/day for men and more than 40 g/day for women) within the past 3 months coupled with elevated aspartate aminotransferase (AST) levels, exceeding alanine aminotransferase (ALT), and total bilirubin >3 mg/dL over the last 3 months. Either a clinically indicated liver biopsy or clinical presentation aligning with alcohol-associated hepatitis was also required. The exclusion criteria included autoimmune liver disease (antinuclear antibody (ANA) greater than 1:320), chronic viral hepatitis, hepatocellular carcinoma, complete portal vein thrombosis, terminal extrahepatic illness, pregnancy, and absence of signed informed consent.

The protocol secured approval from the Ethics Committees at various institutions, including Hôpital Huriez (Lille, France), Universidad Autonoma de Nuevo Leon (Monterrey, Mexico), Hospital Universitario Vall d’Hebron (Barcelona, Spain), King’s College London (London, UK), University of Alberta (Edmonton, Canada), Yale University (New Haven, CT, USA), University of North Carolina at Chapel Hill (Chapel Hill, NC, USA), Weill Cornell Medical College (New York, NY, USA), Columbia University (New York, NY, USA), University of Wisconsin (Madison, USA), VA San Diego Healthcare System (San Diego, CA, USA), and University of California San Diego (La Jolla, CA, USA). Written informed consent was collected from each patient upon enrollment.

#### 2.6.2. Statistics

The results are expressed as the mean ± s.e.m. Three technical replicates were performed for each group.

## 3. Procedure

### 3.1. DNA Isolation from Human Stool

For the isolation of genomic DNA from human stool, the QIAamp Fast DNA Stool Mini Kit (Qiagen, Hilden, Germany) was employed. To optimize the results and enhance the yield of DNA, a protocol modification derived from QIAGEN was implemented. This approach was chosen to ensure the extraction of high-quality DNA from human stool, ultimately contributing to the reliability and accuracy of the downstream analyses.

1. Before starting:
  - Heat the heat block to 95 °C for use in step 4 and 70 °C for use in step 11.
  - Read the instructions from the QIAamp Fast DNA Stool Mini Kit to add absolute ethanol to the Buffer AW1 and Buffer AW2 concentrates. Mix all buffers before use, and redissolve any precipitates in Buffer AL and InhibitEX Buffer by incubating at 37–70 °C. Prepare screw-cap tubes with 2 scoops (0.25 mL) of 0.5 mm beads, and leave them on ice.
2. Weigh 200 mg of stool using a scalpel to scrape bits of the frozen stool sample, and place it in 2 mL screw-cap tubes.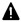 **CRITICAL STEP:** It is important to maintain the sample frozen at all times; keep the sample on ice at all times.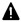 **CRITICAL STEP:** The protocol is optimized for use with 200 mg of stool, but it can also be used with lower or higher amounts. For higher amounts, you need to increase the amount of buffers. For example, weigh the stool sample, and add 10 volumes of Buffer ASL (e.g., add 10 mL InhibitEX to 1 g stool).
3. Add 500 μL of InhibitEX Buffer to each stool sample while keeping it on ice.
4. Use the bead beater at 2000 rpm for 2 cycles of 30 s to homogenize the samples.
5. Using the heat block, heat samples at 95 °C for 5 min.
6. Centrifuge the samples at room temperature (15–25 °C) at 20,000× *g* for 3 min.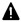 **CRITICAL STEP**: If you see particles in the supernatant, centrifuge the samples again. It is important not to transfer debris.
7. In a new 1.5 mL Eppendorf tube, pipette 20 μL of proteinase K.
8. Transfer 400 μL of the supernatant from step 7 into the 1.5 mL Eppendorf tube containing proteinase K.
9. Pipette 400 μL of AL buffer.
10. Vortex for 15 s.
11. Using the heat block, heat the samples at 70 °C for 10 min.
12. Add 400 μL of absolute ethanol to each tube to the lysate, and vortex for 15 s.
13. Carefully pipette 600 μL of the lysate to the spin column.
14. Centrifuge the samples at room temperature (15–25 °C) at 20,000× *g* for 1 min.
15. Place the spin column in a new 2 mL collection tube, and discard the collection tube that contains the filtrate.
16. Pipette the remaining 600 μL of the lysate to the spin column.
17. Centrifuge the samples at room temperature (15–25 °C) at 20,000× *g* for 1 min.
18. Place the spin column in a new 2 mL collection tube, and discard the collection tube that contains the filtrate.
19. Carefully open the spin column, and add 500 μL of buffer AW1.
20. Centrifuge the samples at room temperature (15–25 °C) at 20,000× *g* for 1 min.
21. Place the spin column in a new 2 mL collection tube and discard.
22. Pipette 500 μL of buffer AW2 in the spin column.
23. Centrifuge the samples at room temperature (15–25 °C) at 20,000× *g* for 6 min.
24. Transfer the spin column into a new labeled 1.5 mL Eppendorf tube, and pipette 50 μL of sterile double-distilled water into the middle of the membrane of the spin column.
25. Incubate 1 min at room temperature.
26. Centrifuge the samples at room temperature (15–25 °C) at 20,000× *g* for 3 min.
27. Discard the spin column, and keep the samples on ice.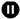 **PAUSE STEP** After collecting all the samples from the centrifuge, they can be stored at −20 °C.
28. Quantification of DNA is accomplished by measuring the absorbance at 260 nm using the Nanodrop.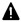 **CRITICAL STEP** The assessment of DNA purity involves calculating the ratio between the absorbance values at 230 nm, 260 nm, and 280 nm. A desirable A260/A280 ratio for pure DNA falls within the range of 1.8 to 2.0, indicating pure DNA. The A260/A230 ratio assesses contaminants, like phenol, salts, and carbs. A ratio above 2.0 suggests minimal contamination.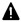 **CRITICAL STEP** To ensure accurate measurements, the absorbance readings at 260 nm should ideally range between 0.1 and 1.0. Maintaining absorbance values within this range is crucial for obtaining reliable and valid quantification results.

### 3.2. Real-Time Quantitative PCR

This protocol outlines the steps for conducting qPCR experiments to amplify bacterial genomic DNA from human stool samples using Sybr Green as the detection method. The primers for the bacterial targets (*E. faecalis*, *E. faecalis CylLs*, and *16S*) were derived from published sequences and are listed in Table 1.

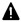
 **CRITICAL STEP** Ensure all pipetting and handling procedures are conducted with appropriate sterile techniques to prevent contamination. Proper controls (negative and positive) should be included in each qPCR run for result validation. Additionally, it is important to verify the specificity of the primers and optimize the primer concentrations, if necessary, to achieve reliable qPCR results.

1. Prepare the qPCR reaction mix for each sample according to the following composition:
  - Sybr Green: 10 μL
  - Primer mix (forward and reverse, 10 μM): 1 μL
  - Extracted DNA (10 ng/μL): 9 μL
2. Mix the components gently by pipetting up and down a few times and vortex 10 s.
3. Distribute the reaction mix into the wells of a qPCR 96-well plate, ensuring proper allocation for samples and controls.
4. Seal the qPCR plate with an optical adhesive cover to prevent contamination during the amplification process.
5. Spin the qPCR plate 10 s at 20,000× *g*.
6. Load the sealed qPCR plate into the ABI StepOnePlus real-time PCR system.
7. Set up the qPCR program on the ABI StepOnePlus system as follows:
  - Initial denaturation: 95 °C for 3 min
  - Amplification (40 cycles)
  - Denaturation: 95 °C for 15 s
  - Annealing and extension: 60 °C for 1 min
  During the amplification cycles, the ABI StepOnePlus system will collect real-time fluorescence data.
8. After the amplification is complete, the system will automatically generate Ct (cycle threshold) values for each reaction, representing the cycle at which the fluorescence signal crosses a predetermined threshold.

### 3.3. Verification of qPCR Product

#### 3.3.1. Melting Curve

The melting curve analysis is crucial for confirming the specificity of the qPCR amplification products. This protocol outlines the steps to perform a melting curve analysis using a real-time PCR instrument:

1. After the completion of the qPCR amplification cycles, initiate the melting curve analysis on the qPCR instrument.
2. Set the temperature range for the melting curve analysis. This typically involves heating the samples from the annealing temperature to a higher temperature, allowing for the DNA to denature.
3. The instrument will measure the fluorescence at each temperature increment as the DNA denatures. The resulting data will be used to generate the melting curve.
4. Once the analysis is complete, review the melting curve graph. Look for distinctive peaks that correspond to the specific DNA products.
5. Compare the melting curve peaks with the expected melting temperatures (Tm) of the target amplicons using the positive and negative controls. This will help confirm the specificity of the amplification.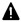 **CRITICAL STEP** Any unexpected peaks, irregularities, or deviations from the expected Tm values should be investigated further, and if necessary, adjustments to the PCR conditions should be made for optimization.

#### 3.3.2. Gel Electrophoresis

Gel electrophoresis is commonly used to visualize and analyze PCR products. This protocol outlines the steps to run a gel electrophoresis for verifying qPCR products.

1. Prepare the agarose gel at 2% using agarose and buffer (TAE). Add SYBR Safe to the gel mix before casting the gel. Cast the gel and allow it to solidify.
2. Mix the qPCR products with loading dye in a 1:1 ratio.
3. Load 3.5 μL of the DNA ladder and 5 μL of the qPCR samples onto the gel wells.
4. Run the gel at 130 V for 20 min.
5. After the electrophoresis is complete, visualize the DNA bands under a UV transilluminator.
6. Compare the size of the DNA bands with the expected sizes of the qPCR products (see Table 1). This helps to verify the presence of the correct amplicons.

### 3.4. Detection of E. faecalis Cytolysin-Positive Strains from Colonies

1. Mix 43 g of *Enterococcosel* Broth in 1 L of MilliQ water for broth preparation, and autoclave the mixture for sterilization.
2. Prepare *Enterococcosel* Agar Plates: Mix 43 g of *Enterococcosel* Broth with 15 g of Agar in 1 L of MilliQ water, and autoclave the mixture for sterilization.
3. Weigh 10 mg of human stool samples and place into 5 mL of *Enterococcosel* Broth.
4. Vortex the mixture until the media is visibly turbulent and well-mixed. Prepare different dilution stocks: 1/1000, 1/10,000, 1/100,000, 1/1,000,000, and 1/10,000,000.
5. Plate 200 µL of the diluted stocks using 4 mm glass sterile beads for equal distribution on the plate.
6. Incubate the plates overnight at 37 °C.
7. Pick colonies that appear on the plates, and perform qPCR using *E. faecalis*-specific and CylLS-specific primers.
8. If the qPCR results indicate positivity for the cytolysin gene, select the same colony from the plate, and incubate it with 5 mL of *Enterococcosel* Broth overnight.
9. Inoculate onto blood agar plates to facilitate the identification of beta-hemolysis caused by the cytolytic toxins released by the bacteria.

### 3.5. Limit of Detection (LOD) and Limit of Quantitation (LOQ)

1. To establish the limit of detection (LOD) and limit of quantitation (LOQ) for the assay, specific criteria were employed. For the detection of *E. faecalis*, a cut-off Ct value of ≤30 was utilized. Similarly, for the detection of CylL_S_, a cut-off Ct value of ≤32 was employed. These Ct value thresholds were determined based on the Ct values from positive and negative controls.
2. To ensure the accuracy and reliability of the assay, two types of controls were used. The positive control for the cytolysin-positive *E. faecalis* strain was obtained from a human stool that was cultured on agar plates to have single colonies. We consistently employ this control to determine variations in qPCR, confirm the reliability of our primers, and validate the quality of our samples.
3. Conversely, a negative control consisting of water was employed to assess and mitigate any potential contamination during the experimental process. This control is vital in confirming the absence of false-positive results.
4. By adhering to these LOD and LOQ thresholds and utilizing appropriate controls, the assay’s sensitivity and specificity were rigorously evaluated, ensuring the validity of the results obtained.

## 4. Expected Results and Discussion

### 4.1. Reproducible DNA Extraction from Human Fecal Samples

To study the reproducible quality of DNA extraction from human fecal samples, the method was assessed through a rigorous experimental design that involved two independent investigators and three separate days of DNA extraction. The goal was to evaluate the consistency and reliability of the DNA extraction procedure, which is a critical step in the downstream molecular analyses.

Fecal samples were processed on three distinct days with each day’s extraction procedure performed by a different investigator (Table 2). This setup allowed for the assessment of both inter-day and inter-investigator variability, contributing to a more comprehensive evaluation of reproducibility.

Quantitative DNA yield analysis was performed using a Nanodrop spectrophotometer. The extracted DNA yield demonstrated minimal variation across the three experimental days and among the different investigators.

Comparing the results obtained by the two independent investigators, a high degree of correlation was evident. This similarity in results confirmed the reliability of the DNA extraction protocol and its resistance to investigator-dependent variability.

### 4.2. Reproducible Ct Values in Real-Time Quantitative PCR from Human Fecal Samples

To ensure the reliability of our findings, we designed a comprehensive experimental setup that involved two independent investigators and qPCR runs performed on three separate days. Additionally, we employed two verification techniques, melting curve analysis and gel electrophoresis, to validate the consistency of qPCR products.

The central focus of our investigation was to establish the consistency of Ct values, a pivotal parameter for quantification in qPCR assays. To evaluate this, the fecal samples were subjected to qPCR on three distinct days with each day’s qPCR procedure conducted by a different investigator (Table 3). This setup facilitated the assessment of inter-day and inter-investigator variability, providing a comprehensive understanding of the assay’s reproducibility.

Quantitative analysis of Ct values was performed on the amplification curves generated during qPCR. Remarkably consistent Ct values were observed across the three experimental days and between the investigators.

To validate the consistency of Ct values, internal controls were included in the qPCR experiments (Table 3). The amplification of reference genes or internal standards showed consistent Ct values across the experimental days and between the investigators. This uniformity indicated that potential variations were not due to the qPCR procedure itself but rather reflective of the actual target DNA levels.

Comparing the results obtained by the two independent investigators, a strong agreement in Ct values was evident. This concordance demonstrated that the qPCR assay’s reproducibility was not influenced by investigator-dependent factors. When evaluating the Ct values across the three experimental days, a consistent trend was observed, indicating that the qPCR assay maintained its reliability and accuracy across multiple days.

**Figure 1:**
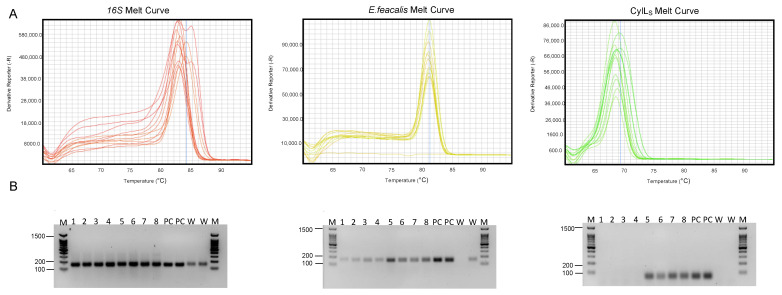
Illustrative verification of qPCR product. (**A**) Melting curves for 16S, *E. faecalis*, and CylL_S_. (**B**) This panel illustrates the gel electrophoresis results of the qPCR product for the 16S rRNA gene (200 bp), *E. faecalis* (142 bp), and *E. faecalis* CylLs (61 bp).

To further bolster our findings, we employed two independent verification techniques: melting curve analysis and gel electrophoresis (Figure 1). The melting curve analysis was conducted to validate the specificity of the qPCR products (Figure 1A). The resulting melting curves consistently displayed single peaks for each target, indicating the absence of non-specific amplification products or primer dimers. This uniformity in melting curves across days and investigators underscored the assay’s specificity and the reliability of Ct value quantification.

Gel electrophoresis (Figure 1B) was used as an additional means to verify the qPCR products. The amplification products were resolved on an agarose gel, and consistent band patterns were observed across all samples. The absence of multiple bands or smearing indicated that the qPCR products were of high quality and lacked contamination or degradation. This verification step reinforced the reliability of the qPCR process.

Furthermore, the practical application of the limit of detection (LOD) and limit of quantitation (LOQ) criteria was demonstrated in evaluating the assay’s performance. Following the analysis of the entire sample set, the striking quality of the melting curve profiles was evident, affirming the integrity of the qPCR products. Applying the LOD and LOQ criteria underscored the practical significance of our study. We employed specific criteria to establish the LOD and LOQ for our assay. For *E. faecalis* detection, a cut-off Ct value of ≤30 was employed, while for CylL_S_, a cut-off Ct value of ≤32 was used. These thresholds were meticulously determined based on their correlation with the presence of the respective targets.

### 4.3. Detection of E. faecalis Cytolysin-Positive Strains from Colonies

In this study, we developed a comprehensive protocol tailored for the precise identification of cytolytic *E. faecalis* strains derived from colonies extracted from human fecal samples.

**Figure 2:**
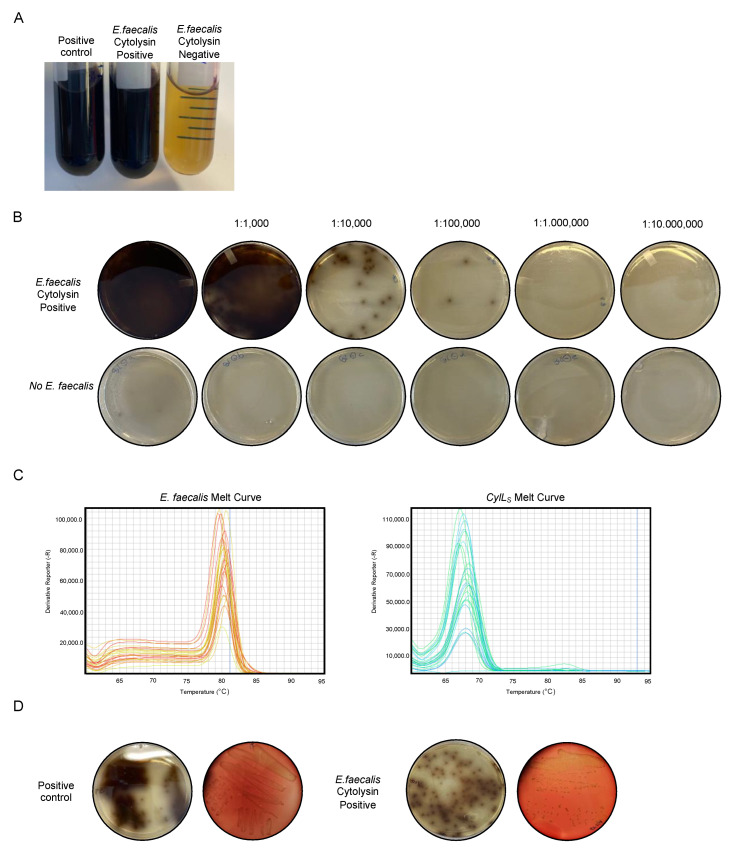
Representative outcomes in the detection of *E. faecalis* cytolysin-positive strains from colonies. (**A**) The results post-overnight culture using *Enterococcosel* Broth. (**B**) Dilutions on *Enterococcosel* agar plates. (**C**) Melting curve outcomes following qPCR for *E. faecalis* and CylL_S_. (**D**) Results on blood agar using positive controls or samples positive for *E. faecalis* cytolysin presence.

To ensure an ideal growth environment for *E. faecalis* strains, we prepared and sterilized *Enterococcosel* Broth and *Enterococcosel* Agar Plates. By precisely weighing and introducing human fecal samples into *Enterococcosel* Broth, we initiated bacterial growth (Figure 2A). As observed in the results, only patients positive for *E. faecalis* cytolysin exhibited the same characteristics as the positive control. The implementation of diverse dilution stocks allowed for proper colony growth and facilitated effective isolation. Following this, we plated the diluted samples using sterile beads onto Enterococcosel agar plates, which subsequently led to incubation and the emergence of distinct colonies. As evident in Figure 2B, only patients positive for *E. faecalis* cytolysin displayed the presence of colonies.

Upon the appearance of colonies, a qPCR analysis was carried out, utilizing *E. faecalis*-specific and cytolysin S-specific primers. This molecular approach was specifically designed to target strains harboring the cytolysin gene. Positive signals within the qPCR analysis prompted the selection of the same colony (Figure 2C), followed by further incubation in *Enterococcosel* Broth. Subsequently, we proceeded with inoculation onto blood agar plates, thereby allowing for the observation of beta-hemolysis—an essential indicator of cytolytic toxins released by the bacteria (Figure 2D).

For the purpose of future analysis, all strains—regardless of their cytolytic attributes—were thoughtfully preserved. This was achieved by preparing glycerol stocks at a 50% concentration, originating from the cultured strains. These glycerol stocks proved to be invaluable long-term storage solutions, ensuring the retention and future retrieval of the strains for subsequent research endeavors.

In conclusion, here we developed a standardized real-time quantitative polymerase chain reaction (RT-qPCR) assay to detect and quantify cytolysin in fecal samples from patients with alcohol-associated hepatitis. The diagnostic assay allows for the identification of cytolysin-positive patients who can be selected for clinical trials.

## Figures and Tables

**Table 1:** Primers used in this protocol.

Gene	Primer	Sequence	Product Size (bp)
*E. faecalis* [13]	F R	5′-CGCTTCTTTCCTCCCGAGT-3′ 5′-GCCATGCGGCATAAACTG-3′	142
*E.faecalis* Cytolysin small subunit (CylL_S_) [14]	F R	5-GTAAAATAAGTAAAATCAAGAAAACTATTACTC-3 5-CAAAAGAAGGACCAACAAGTTCTAATT-3	61
16S rRNA [15]	F R	5-GTGSTGCAYGGYTGTCGTCA-3 5-ACGTCRTCCMCACCTTCCTC-3	200

**Table 2:** DNA quantification inter-day and inter-investigator quality.

Investigator 1						Investigator 2				
Replicate	Sample	DNA (ng/uL)	260/280	260/230		Replicate	Sample	DNA (ng/uL)	260/280	260/230
**1**	1A	153	2.05	1.65		**1**	1B	236.3	2.1	1.59
	2A	92.2	2.1	1.88			2B	86	2.1	1.73
	3A	84.5	2.12	2.3			3B	492.6	2.1	2.17
	4A	292	1.98	1.03			4B	284	2.08	1.92
										
**2**	1A	409.4	2.09	1.46		**2**	1B	152.9	1.93	0.92
	2A	114.8	2.1	1.9			2B	902.7	1.97	1.8
	3A	1159.1	1.94	1.36			3B	1413.3	2.16	2.29
	4A	480.1	2.03	1.28			4B	272	1.8	0.83
										
**3**	1A	232.4	2.11	1.47		**3**	1B	289.3	2.01	1.24
	2A	151.6	2.15	1.88			2B	142	2.15	1.73
	3A	2045.3	2.14	2.26			3B	2200.2	2.13	2.28
	4A	246.8	2.07	1.14			4B	135.1	2.09	1.373

**Table 3:** Ct value results inter-day and inter-investigator reproducibility.

Investigator 1					Investigator 2				
Replicate	Sample	Cт *E. faecalis*	Cт CylLS	Cт 16S	Replicate	Sample	Cт *E. faecalis*	Cт CylLS	Cт 16S
**1**	1	36.798	Undetermined	12.392	**1**	1	30.374	Undetermined	10.263
	1	Undetermined	36.919	12.268		1	30.318	Undetermined	10.708
	2	35.089	Undetermined	13.145		2	31.379	34.200	11.423
	2	35.911	Undetermined	13.207		2	29.385	Undetermined	11.999
	3	21.983	28.645	12.453		3	17.598	26.769	7.001
	3	21.936	28.482	12.504		3	17.227	26.388	7.521
	4	24.393	26.505	12.906		4	23.211	Undetermined	11.728
	4	24.493	26.370	12.939		4	22.555	26.952	11.173
	Positive control 1	11.945	15.558	12.026		Positive control 1	11.725	15.558	9.965
	Positive control 2	11.977	15.461	12.070		Positive control 2	12.804	15.461	7.696
	Negative Control	Undetermined	Undetermined	31.529		Negative Control	33.474	Undetermined	36.040
	Negative Control	Undetermined	36.830	30.843		Negative Control	37.150	Undetermined	31.099
**2**	1	37.005	Undetermined	12.901	**2**	1	33.814	37.106	13.160
	1	36.418	Undetermined	12.936		1	33.641	33.427	13.903
	2	33.991	Undetermined	12.123		2	34.525	34.890	13.935
	2	35.720	Undetermined	12.175		2	35.179	36.381	12.222
	3	19.944	27.239	12.200		3	22.255	26.421	12.411
	3	19.883	27.256	11.978		3	22.532	26.788	11.842
	4	22.651	24.848	12.669		4	28.119	26.855	13.983
	4	22.640	24.868	12.700		4	27.506	27.175	13.882
	Positive control 1	11.704	15.611	12.396		Positive control 1	14.212	13.181	30.131
	Positive control 2	11.411	15.614	12.505		Positive control 2	13.993	13.557	29.480
	Negative Control	Undetermined	Undetermined	31.164		Negative Control	35.373	Undetermined	12.468
	Negative Control	36.216	Undetermined	31.511		Negative Control	35.931	Undetermined	12.427
**3**	1	37.113	Undetermined	13.059	**3**	1	32.038	35.604	15.222
	1	36.873	Undetermined	12.919		1	34.958	Undetermined	15.650
	2	33.919	36.075	12.697		2	33.430	34.193	15.333
	2	Undetermined	35.888	12.664		2	33.989	Undetermined	14.092
	3	18.491	27.679	11.946		3	27.323	30.231	15.636
	3	18.555	27.459	11.837		3	24.076	28.918	14.322
	4	22.607	26.641	14.437		4	27.831	25.903	16.883
	4	22.582	26.562	14.497		4	30.392	28.336	17.249
	Positive control 1	9.402	15.272	13.242		Positive control 1	13.046	12.979	13.487
	Positive control 2	9.408	15.155	13.265		Positive control 2	13.175	12.901	13.332
	Negative Control	32.194	37.043	30.759		Negative Control	36.891	32.877	30.982
	Negative Control	Undetermined	36.288	30.606		Negative Control	34.509	36.565	30.959

Samples 1–2: DNA extracted from stool negative for cytolysin producing *E. faecalis*. Samples 3–4: DNA extracted from stool positive for cytolysin producing *E. faecalis*. Positive control: cultured cytolysin positive *E. faecalis*. Negative control: water.

## Data Availability

Data is contained within the article.

## Abbreviations

ALD	Alcohol-Associated Liver Disease
ALT	Alanine Aminotransferase
AST	Aspartate Aminotransferase
Ct	Cycle Threshold
CylLS	Cytolysin small subunit
LOD	Limit of Detection
LOQ	Limit of Quantitation
qPCR	Quantitative Polymerase Chain Reaction
RT-qPCR	Real-Time Quantitative Polymerase Chain Reaction
TAE	Tris-Acetate-EDTA
Tm	Melting Temperature
UV	Ultraviolet
